# Asymmetric Diffusion Metasurface-Based Lensless Monitoring and Uniform Illumination Systems for Optical Lithography

**DOI:** 10.34133/research.1197

**Published:** 2026-03-23

**Authors:** Guangbiao Wang, Yanhua Shen, Yazhi Pi, Wenchao Kong, Yun Lai, Xu Ma, Yan Zhou, Fan Li, Zizheng Cao, Lei Wang, Shaohua Yu

**Affiliations:** ^1^ School of Electronics and Information Technology, Sun Yat-Sen University, Guangzhou 510275, China.; ^2^ Peng Cheng Laboratory, Shenzhen 518055, China.; ^3^ Department of Electronic and Information Engineering, Harbin Institute of Technology (Shenzhen), Shenzhen 518055, China.; ^4^ National Laboratory of Solid State Microstructures, School of Physics, and Collaborative Innovation, Center of Advanced Microstructures, Nanjing University, Nanjing 210093, China.; ^5^ Key Laboratory of Photoelectronic Imaging Technology and System of Ministry of Education of China, School of Optics and Photonics, Beijing Institute of Technology, Beijing 100081, China.; ^6^ College of Integrated Circuits, ZJ U-Hangzhou Global Scientific and Technological Innovation Center, Zhejiang University, Hangzhou 310027, China.; ^7^ Shanghai Institute for Advanced Study, Zhejiang University, Shanghai, China.

## Abstract

The long-standing trade-off between illumination performance (such as uniformity) and optical system complexity has hindered the development of compact lithographic tools. Here, we overcome this limitation by introducing a metasurface-enabled lensless illumination system that seamlessly combines light homogenization and monitoring capabilities. Using an asymmetric diffusion metasurface integrated with intelligent optimization algorithms, we achieve precise light field control and self-adaptive feedback without relying on conventional lens assemblies. When deployed on a self-developed large-field lithography platform, the system demonstrates uniform ultraviolet illumination with nonuniformity below 3% and successfully exposes patterns across a range of feature sizes. A gray-level to power-level mapping algorithm enables real-time assessment of light-emitting diode performance, providing diagnostic alerts and adaptively reconfigurable exposure configurations to circumvent defective emitters. This synergy between metasurface optics and algorithmic intelligence establishes a new paradigm for compact, low-cost, and scalable lithographic illumination, offering a pathway toward next-generation intelligent manufacturing systems.

## Introduction

Lithography is the cornerstone of semiconductor manufacturing [1–5], and the illuminator in the lithography system is a key factor in balancing patterning precision, production efficiency, and equipment scalability [6,7]. In conventional lithography systems, 2 core functional modules—namely, the homogenization unit for uniform mask illumination and the monitoring unit for energy stability and deviation correction—are typically implemented as independent optical subsystems [8]. For over half a century, conventional homogenization units relied on the Köhler illumination principle to suppress source nonuniformity [9,10]. They commonly employ cascaded lenses [11,12], microlens arrays (MLAs) [13–16], or light integrator rods to achieve the illumination uniformity required for high-fidelity lithography (below 3% for mask aligners) [7,17]. While effective, these approaches suffer from inherent limitations, including high costs, excessive bulk, and suboptimal maintainability. In particular, integrator rods based on internal reflections exhibit low transmittance, bulky dimensions, absence of polarization-maintaining capability, and limited emission angle, making them unsuitable for compact and high-performance illumination systems [17]. Likewise, MLA-based solutions often require a coupling condenser lens group and a tandem MLA integrator configuration, which further increases system complexity and alignment burden [18]. Recent approaches have leveraged the freeform optics to achieve high pattern fidelity [19–21]. Nonetheless, these methods face some constraints, including the complex design, manufacturing challenge, and high cost. For the real-time monitoring unit, conventional solutions depend on additional beam-splitting optics and energy-sensing components, which further increase the system complexity and cost [7,8]. Such single-sided illumination architectures rely on separate optical paths to independently manipulate reflected and transmitted light, which inevitably increases system complexity, volume, and alignment sensitivity. These drawbacks limit their applicability to cost-sensitive industrial and consumer applications such as large-area flexible electronics or on-chip microfabrication [22–24].

The rapid development of optical metasurface technology and computing power offers new opportunities to improve the traditional illumination systems. By locally tailoring the optical response of nano-structured elements to control the phase, amplitude, and polarization across a single interface, these ultrathin artificial materials can precisely manipulate the light waves at nanoscale. This capability outperforms the conventional paradigm of bulky refractive optics, offering a scalable pathway to miniaturize and integrate the complex optical functions into a flat single-layer device. From aberration-free metalenses [25,26] and vectorial holography [27,28] to polarization encryption [29,30] and computational imaging [31], metasurfaces open unprecedented opportunities in photonics, sensing, and quantum information processing [32–41]. The compatibility of the metasurface with the standard semiconductor nanofabrication techniques further makes it a promising technology for the next-generation optoelectronic systems. Despite the advent of metasurfaces, a monolithic architecture that combines optical homogenization and monitoring remains unrealized. To the best of our knowledge, no prior work has demonstrated such an integrated design within a lensless framework, nor has any implementation been successfully validated on industrial-grade lithography systems.

Here, we propose a dual-sided metasurface architecture that integrates illumination homogenization and real-time monitoring into a single ultracompact platform. Unlike conventional single-sided designs, the proposed structure exploits asymmetric scattering responses on the 2 sides of the metasurface, enabling independent manipulation of reflected and transmitted light fields. This dual-sided strategy allows uniform illumination to be achieved through the reflection channel, while the transmission channel provides direct access to monitoring information, without introducing additional optical components. Based on this concept, we develop a lensless illumination system based on asymmetric diffusion metasurfaces (ADMs); this system integrates homogenization and monitoring functions in a single architecture. Our ADMs function as bidirectional anisotropic elements with independently tunable diffusion reflection for uniform exposure (reflection side) and the normal transmission for ultraviolet light-emitting diodes (UV LEDs) state monitoring (transmission side). We use a genetic algorithm to optimize the illumination configuration (achieving illumination nonuniformity [INU] below 3%), and use a gray-level to power-level mapping algorithm (GL-PLMA) to implement real-time fault detection (achieving 100% fault accuracy). The system is then validated on a self-developed large-field proximity lithography platform. We successfully expose patterns with feature sizes down to 2.5 μm and demonstrate uninterrupted operation even with some faulty LEDs, thus eliminating the need for costly module replacement. These results highlight a compact, scalable, and cost-effective approach toward next-generation lithographic illumination systems that integrate multiple functionalities within a single planar optical element.

## Results

### System model

**Fig. 1.**
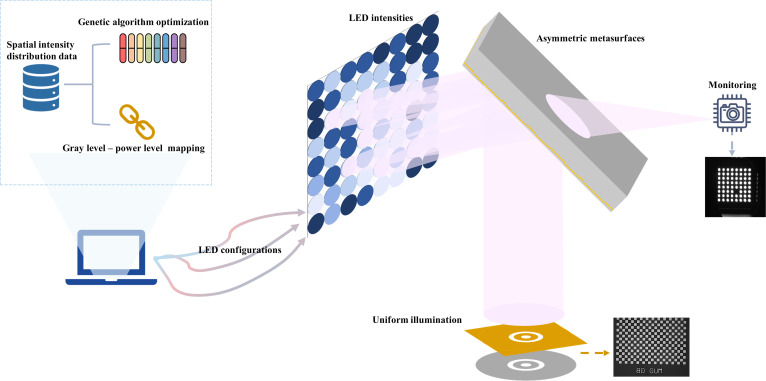
Schematic diagram of the proposed novel lithographic illumination system relying on asymmetric diffusion metasurfaces (ADMs) and intelligent algorithms. With the metasurfaces, the system integrates uniform illumination on the reflection side with real-time monitoring on the transmission side.

As illustrated in Fig. 1, we propose a novel lithographic illumination system that integrates monitoring and homogenization within a single architecture. The system is based on a highly asymmetric diffusion via macroscopic disordered ADMs. For homogenization, spatial intensity distribution data of UV LEDs under various conditions are precollected, based on which the illumination configuration is optimized through a genetic algorithm to satisfy the uniformity requirement. By combining the emissions of multiple LEDs reflected by the metasurfaces, uniform exposure at the wafer plane is achieved. For real-time monitoring, images on the transmission side are captured by a camera, and the GL-PLMA is used for monitoring the operating states of individual LEDs in real time. This system model inherently incorporates the 4-step workflow: (a) the fundamental source database is built via the spatial intensity acquisition system; (b) the exposure-monitoring correspondence is realized by the GLPMA; (c) the genetic algorithm optimizes LED configurations; and (d) optimized configurations are fed into the source control module for real-time operation. The system comprises 3 core components: ADMs, the GL-PLMA for monitoring, and the genetic algorithm-based optimization for uniform exposure, which will be described in details next.

### Asymmetric diffusion metasurfaces

The ADMs employed in this work serve as an asymmetric beam splitter, with independently tunable reflection and transmission characteristics. Its design leverages 2 particles’ interference to suppress the specular reflection. By carefully controlling the thickness of the binary reflecting metal or dielectric patches and their embedded depths within the substrate, the transmitted beams through the 2 meta-atoms exhibit a vanishing phase difference, while the reflected beams maintain a phase difference of π. Randomized arrangements of the binary unit cells on a large-area metasurface give rise to direction-dependent diffusion, manifesting as strong reflective diffusion accompanied by weak transmissive diffusion within a broad or specified frequency window.

**Fig. 2.**
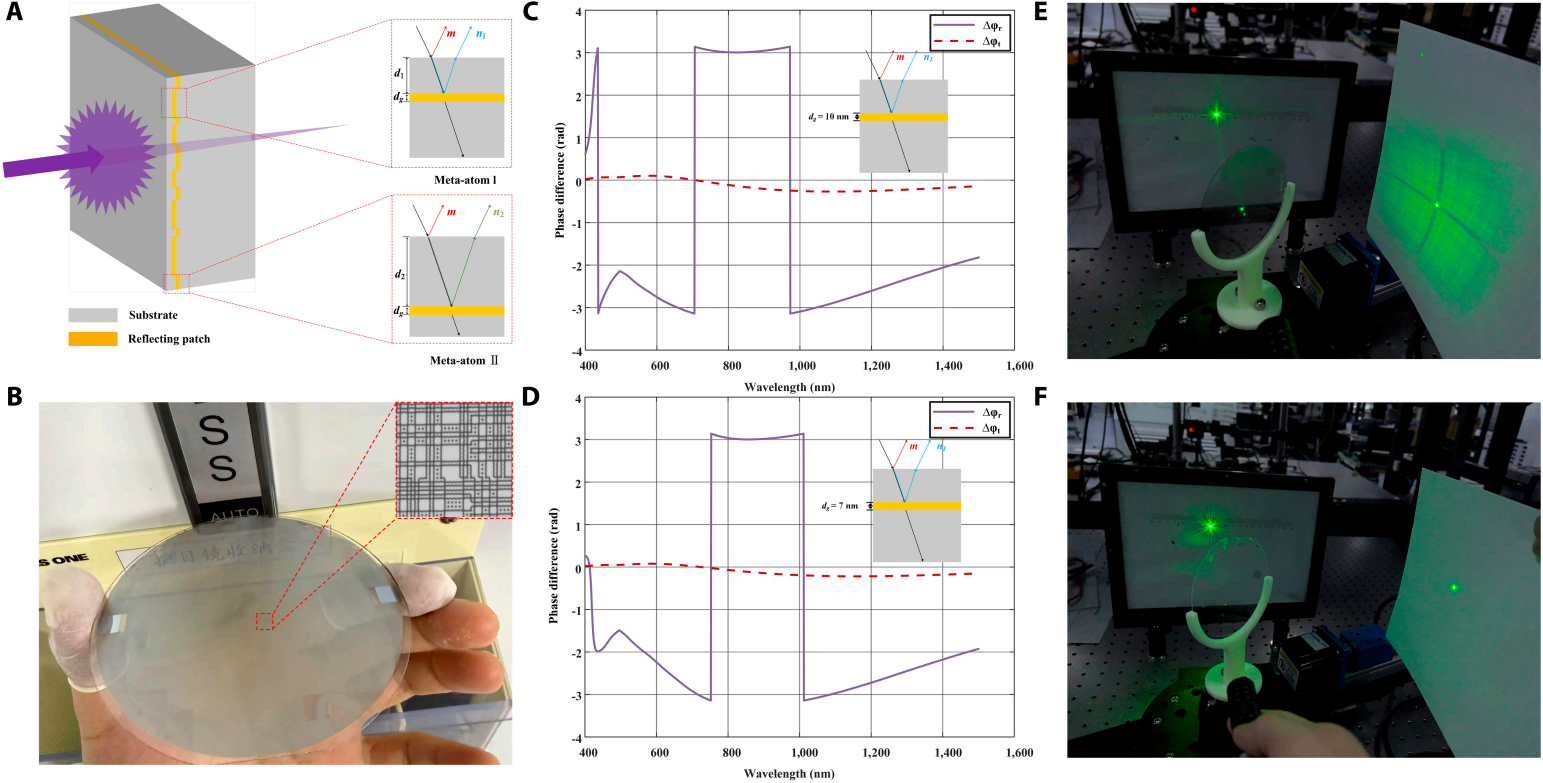
Structural model and experimental demonstration of ADMs. (A) Schematic illustration of the asymmetric diffusion model induced by meta-atom interference. The 2 meta-atoms are composed of reflective patches with a thickness of *d*_g_ (yellow), embedded at depths *d*_1_ and *d*_2_ below the surface of the dielectric substrate (gray). (B) Photograph of the metasurfaces sample deposited on a 4-inch glass wafer and a zoom-in photograph of its unit showing the random distribution. (C and D) Phase differences in reflection ($\Delta \varphi_{r}$) and transmission ($\Delta \varphi_{t}$) for 2 meta-atoms constructed by Aurum patches of *d*_g_ = 10 nm and 7 nm, respectively. (E) The light field distribution after laser illumination of the metasurface sample shows that the transmitted light remains well-focused, while the reflection exhibits pronounced asymmetric diffusion, demonstrating the coexistence of asymmetric diffusion reflection and normal transmission. (F) The test results of laser illumination on a smooth glass substrate show that both transmission and reflection correspond to normal transmission and specular reflection.

As illustrated in Fig. 2A, the 2 fundamental meta-atoms are planar optical elements. Following the meta-atom configuration reported in a previous work [42], when the light impinges on the first surface, part of it is reflected, as illustrated by the ray *m* in the figure, while the other part is refracted into the interface with the reflective medium layer, where it undergoes further reflection and refraction. The reflected component then returns to the incident space through the first surface, as represented by the rays *n*_1_ and *n*_2_. For simplicity, only the first- and second-order terms of these multiple reflections are considered. Meanwhile, the transmitted light propagates toward the second surface and exits via refraction. Because the 2 meta-atoms provide identical optical path lengths for the transmitted fields, their transmission phases remain matched. In contrast, the reflective responses of the unit cells originate from the combined contributions of the dielectric interface and the embedded reflective patches. The interface-related contribution is common to both meta-atoms and is governed by the refractive index of the substrate, whereas the patch-induced contributions depend sensitively on the vertical embedding depths. By appropriately selecting the depths *d*_1_ and *d*_2_, patch-related responses can be engineered to be either in phase or out of phase with the interface contribution at the target frequency, thereby producing a reflection-phase contrast between the 2 meta-atoms. The substrate can be composed of isotropic homogeneous materials (e.g., glass and fused silica) or anisotropic materials (e.g., birefringent media). The reflective patches may be fabricated from metals (e.g., Au and Ti) or dielectric materials. Optical coatings on the surface allow precise tuning of the transmission and reflection, thus controlling the relative intensity of the illumination (reflected light) and monitoring (transmitted light) channels. The transmission can be substantially enhanced by reducing the thickness of the metallic patches or replacing them with the dielectric counterparts.

The unit is designed by arranging the 2 meta-atoms shown in Fig. 2A into an 800×800 random pattern, covering an area of 0.72mm×0.72mm. The unit is eventually repeated to form a structure consisting of a 7-nm-thick gold layer (with *d*_g_ = 7 nm, *d*_1_ = 73 nm, and *d*_2_ = 115 nm) deposited on a 4-inch glass wafer substrate, as shown in Fig. 2B. The phase differences in reflection ($\Delta \varphi_{r}$) and transmission ($\Delta \varphi_{t}$) for the 2 meta-atoms constructed by Aurum patches of *d*_g_ = 10 and 7 nm are illustrated in Fig. 2C and D, respectively. It can be observed that the transmitted beam through the 2 meta-atoms exhibits phase difference cancellation across a broad bandwidth, while the reflected beam maintains a π phase difference at certain frequencies and bandwidths. Experimental validation with a 532-nm laser demonstrates that the metasurfaces achieve unidirectional diffuse reflection while preserving normal transmission, as shown in Fig. 2E. By contrast, as shown in Fig. 2F, the unstructured glass substrate exhibits conventional transmission and reflection with no discernible diffusion effects, confirming the metasurface-induced asymmetry.

### Monitoring: Gray-level to power-level mapping algorithm

**Fig. 3.**
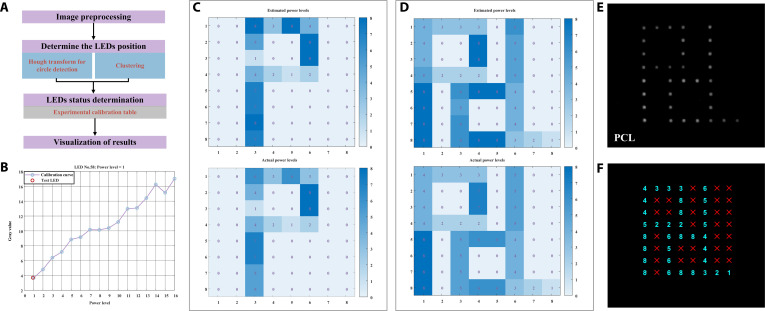
Implementation workflow and experimental results of the monitoring gray-level to power-level mapping algorithm (GL-PLMA). (A) Flowchart of the monitoring algorithm, including the steps of image preprocessing, light-emitting diode (LED) position determination, LED status determination, and visualization of results. (B) Gray level-power level mapping curve of the No. 58 LED. (C) Estimated (top) and actual (bottom) results for the LED array with “P” shape illumination at different power levels. (D) Estimated (top) and actual (bottom) results for the LED array with “PCL” shape illumination at different power levels. (E) Experimental image of the LED array with “PCL” shape illumination at different power levels. (F) Visualization of the LED [in (E)] operational status; red X for deactivated or faulty LEDs, and cyan numerical labels for power levels.

The light emitted from UV LED array transmits through the metasurfaces. By placing a camera at the monitoring side and leveraging a pre-established database of spatial light intensity distributions for different UV LED parameters, the relationship between the captured grayscale values and the individual UV LED power levels can be determined. This enables real-time monitoring of the light source status. Given the known correspondence between the light source parameters and the exposure-side intensity distribution, the field distribution at the exposure plane can also be inferred from the monitoring-side image, which allows real-time supervision of uniform illumination and the active control of homogenization performance. To determine the status of the entire LED array, we apply an image-processing algorithm comprising the following steps, as illustrated in Fig. 3A.

Firstly, image preprocessing. Images are processed by first converting to grayscale, denoising, and correcting background variations. Secondly, determining the position of the LEDs. The centers and radii of the illuminated LEDs were automatically identified using the Hough circle transform, and clustering of the detected bright LEDs was performed to determine the row and column numbers and spacing, allowing reconstruction of the complete array coordinates. Thirdly, determining the status of the LEDs. Mean intensities within these regions of interest for each LED are compared to thresholds to determine on/off states and mapped to power levels using the calibration table. Finally, visualization of the results. The results are then annotated on the images and exported as structured datasets, providing a pixel-resolved feedback mechanism for monitoring and illumination homogenization.

By preacquiring images of UV LEDs at different power levels and calculating their grayscale values, we established a correspondence between gray levels and LED power levels (Fig. 3B), together with a calibration table for status determination. By calculating the gray value of each LED region under test and comparing it with the calibrated reference levels, the operating power level is determined as the closest match. To validate the monitoring functionality, we test a “P”-shaped illumination pattern, in which LEDs at different positions are assigned different power levels, with several LEDs intentionally turned off (simulating device failure). As shown in Fig. 3C, the upper panel illustrates the estimated power-level distribution obtained using the proposed method, while the lower panel presents the actual illumination distribution. The results demonstrate a power-level identification accuracy of 90.62% and a fault-LED detection accuracy of 100%, with only minor fluctuations in level recognition for some LEDs. A second validation experiment is performed using a “PCL” illumination pattern. The results, shown in Fig. 3D, indicate a power-level identification accuracy of 82.81% and a fault detection accuracy of 100%. The reduction in power-level accuracy is primarily attributed to light-source instability, camera acquisition noise, and environmental perturbations. Figure 3E presents the raw LED array image corresponding to the “PCL” illumination pattern, highlighting the different power levels assigned to individual LEDs. Figure 3F shows the algorithm-based recognition and visualization results, where faulty LEDs in the off-state are clearly and unambiguously identified with a red cross. The monitoring algorithm accurately reflects the real-time status of each LED. Faulty LEDs are correctly identified, enabling the optimization algorithm to generate an equivalent uniform illumination scheme that excludes malfunctioning LEDs, thereby avoiding the need to halt operation and replace the entire light source due to a single LED failure.

### Uniform illumination based on genetic algorithm

To determine a light source configuration that achieves the target uniformity distribution, i.e., the optimized illumination configuration, an initial dataset is acquired by measuring the spatial intensity distributions at the exposure plane (10cm×10cm) for individual LEDs operated independently at different power levels after reflection by the metasurfaces. This produces a data library of 1,024 intensity matrices containing the spatial intensity distributions for 64 LEDs at 16 power levels.

As shown in Fig. S1, a genetic algorithm is employed to search for the optimal solution [43,44]. The LED data from the database are encoded sequentially. The algorithm creates a random or given initial population. The objective function is defined as the INU of the incoherently superimposed light intensity at the exposure plane, which is defined in Eq. (1). Equation (2) is the illumination uniformity (IU) [45].

$$\mathrm{INU}=\frac{P_{\max}-P_{\min}}{P_{\max}+P_{\min}}\times 100\%,$$ (1)

$$\mathrm{IU}=\left(1-\frac{P_{\max}-P_{\min}}{P_{\max}+P_{\min}}\right)\times 100\%.$$ (2)

The fitness score of each chromosome is then evaluated and the population is sorted accordingly. The algorithm checks whether the termination criteria, either a maximum number of generations or a target fitness value, have been satisfied. If not, tournament selection is employed, a subset of individuals is chosen at random, and the fittest individual within this subset is selected as a parent. A specified crossover fraction determines the proportion of the population, excluding elite offspring, that results from crossover. Uniform mutation is applied as the variation scheme. A new population is then generated, and fitness values are recalculated. This process is iteratively repeated until the termination conditions are met. Through iterative selection, crossover, and mutation operations, the algorithm evolved toward a globally optimal uniformity distribution, producing a combination of LED pixel parameters capable of realizing the target homogenized illumination.

In the event of light source anomalies, such as individual LED aging or power instability, deviations between the precomputed illumination configurations and the real-time monitored output trigger feedback to the core algorithm. The system can then switch to an alternative illumination configuration that achieves equivalent uniformity and bypasses the problematic LEDs whenever possible. If repeated switching fails, the core algorithm assists in identifying malfunctioning LEDs and notifies the user to maintenance or replacement of the light source. To minimize the latency in practical operation, the system can precompute and store optimized LED configurations for all faulty LEDs scenarios. Once a fault is detected, the system only needs to identify the faulty LEDs and apply the corresponding precomputed configuration, which requires only negligible time for fault recognition and communication.

### Simulation results

**Fig. 4.**
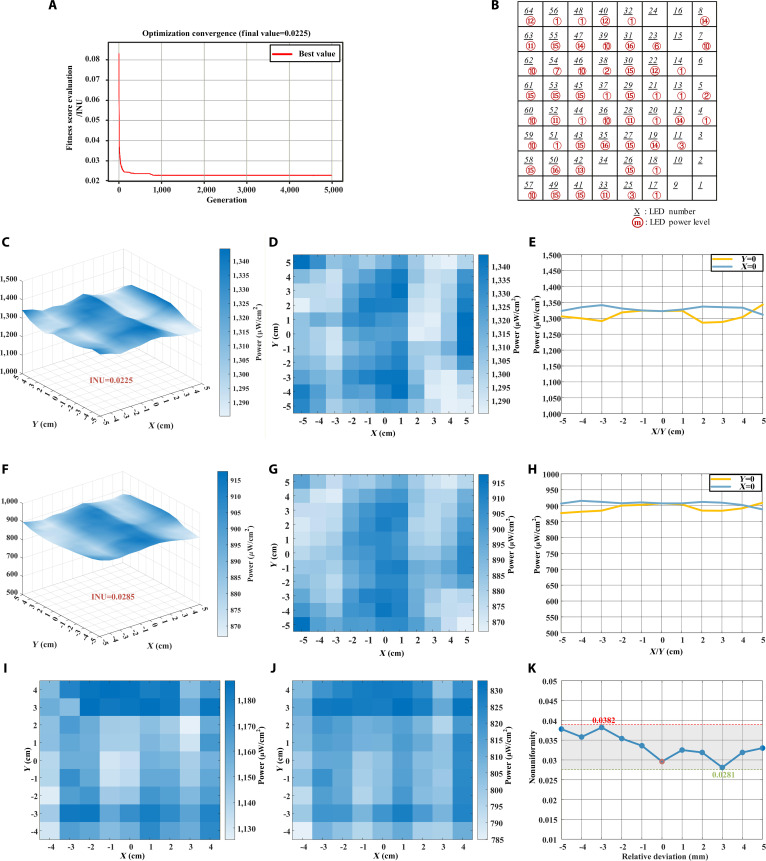
Optimization and experimental results of the LED illumination configuration using a genetic algorithm. (A to E) Optimization results: (A) The convergence of the objective function. (B) The optimized illumination configuration. (C) 3D power distribution. (D) 2D map. (E) The cross-sectional power distribution in (D). (F to H) Experimental results: (F) 3D power distribution. (G) 2D map. (H) The cross-sectional power distribution (G). (I to K) Experimental results in another long-distance (10 cm) application scenario, and the tolerance results of nonuniformity with respect to distance: (I) 2D map of the simulation light power distribution. (J) 2D map of the experimental light power distribution. (K) Scatter plot of illumination nonuniformity with respect to displacement of ±5 mm from the reference sampling plane.

Following the above principles, we performed numerical simulations in Python, where the genetic algorithm was implemented using the Distributed Evolutionary Algorithms in Python toolbox. The simulation parameters are summarized in Table S1. The optimization variables were defined as the indices of the intensity data matrix at the detection plane corresponding to each LED and its power level. The simulations were carried out on a desktop computer (AMD Ryzen 7 8845H, 32 GB RAM). Under the GA configuration of population size 1,200, tournament selection size 3, elitism preserving 10% best individuals, crossover probability 0.85, 2-point crossover, mutation probability 0.3 with uniform mutation, and maximum 5,000 generations, the optimization was completed in 521 s after 5,000 generations. The simulation results are presented in Fig. 4A to E.

Figure 4A shows the convergence of the objective function, where the nonuniformity rapidly decreased and reached the global optimum of 2.25% within the first 1,000 generations. By decoding the optimal solution into the corresponding LED indices and power levels, we obtained the optimized illumination configuration, as shown in Fig. 4B, in which black underlined numbers denote the LED indices and red circled numbers denote the assigned power levels. The resulting light power distribution on the detection plane is illustrated in Fig. 4C (3-dimensional [3D] surface plot) and Fig. 4D (2-dimensional [2D] map), yielding a maximum irradiance of 1,344.3μW/cm^2^, a minimum of 1,285.1μW/cm^2^, and a mean of 1,312.8μW/cm^2^, with a nonuniformity of 2.25%. Figure 4E shows the cross-sectional power distribution at the center of the *XY* plane (*X* = 0, *Y* = 0).

### Experiment results

**Fig. 5.**
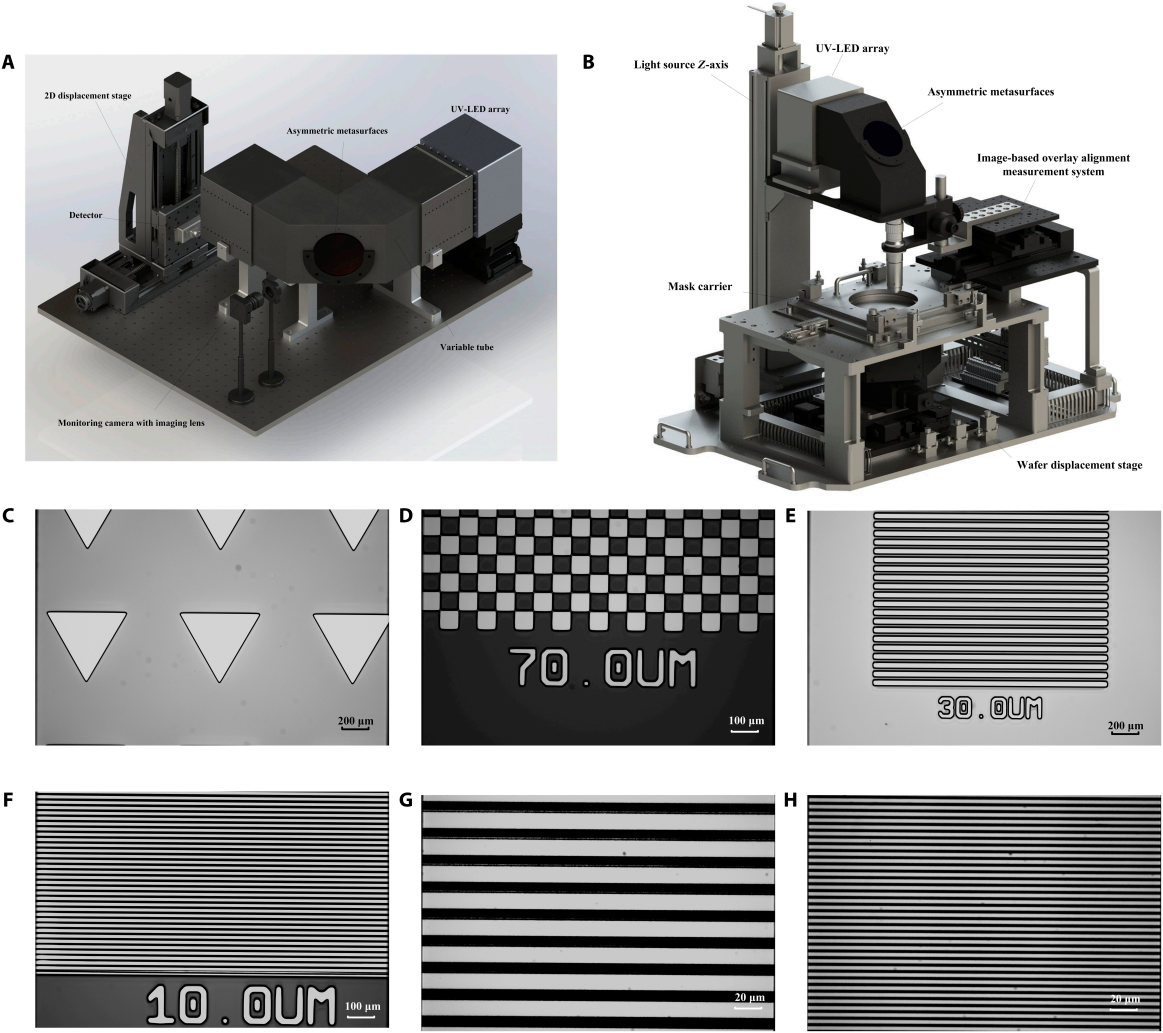
Photograph of the spatial light intensity acquisition system, the self-developed proximity lithography platform, and the developed patterns on the self-developed proximity lithography platform with the proposed illumination system. (A) Photograph of the spatial light intensity acquisition system. (B) Photograph of the self-developed wafer-scale, large-field proximity lithography platform. (C to H) Photograph of the developed patterns on the self-developed proximity lithography platform with the proposed illumination system: (C) 600-μm equilateral triangle at 140 mJ/cm^2^; (D) 70-μm grid at 140 mJ/cm^2^; (E) 30-μm line pair at 150 mJ/cm^2^; (F) 10-μm line pair at 140 mJ/cm^2^; (G) 10-μm line pair at 170 mJ/cm^2^; and (H) 2.5-μm line pair at 170 mJ/cm^2^.

To validate the feasibility of the proposed lensless monitoring and uniform illumination lithography system based on ADMs and intelligent algorithms, we conducted experimental tests on the spatial light intensity acquisition system and a self-developed wafer-scale, large-field proximity lithography platform. As illustrated in Fig. 5A, we constructed a spatial light intensity acquisition system for the LED array to establish the database. The system consists of a UV LED array (as shown in Fig. S2), an adjustable-length optical tube, an ADMs window, a UVA LED digital probe (Linshangtech, LS129-UVALED-X3), a high-precision 2D displacement stage (OMTOOLS, OMT-24101109DK), and a monitoring camera (THORLABS, CS165MU) equipped with an imaging lens (THORLABS, MVL4WA). In addition, a square slot is reserved in the tube near the light source side for inserting brightness enhancement films [46], which improve energy utilization and control the angular distribution of the light. On the side near the detector, a light shaping filter plate can be mounted to tailor the angular spectrum of the illumination [12]. The light emitted by the LEDs propagates through the tube, with part of the beam reflected by the ADMs toward the detector plane and the other part transmitted through the metasurfaces to the monitoring camera. The tube length can be adjusted according to the requirements of different situation in practical applications. The data acquisition process is as follows: each LED is turned on individually in sequence starting from the first one, with the brightness level beginning from the first level. The digital detector is moved within a 10cm×10cm area at 1-cm intervals, scanning in an S-shaped pattern to record the power value of each LED at the given level. The average of 4 measurements is taken as the power value at each point.

In one experiment, the detector plane was positioned 10 mm from the tube’s light-emitting surface. Data were collected at 1-cm intervals within a 10cm×10cm area. The optimized results obtained from the genetic algorithm simulation, namely, the LED driving configuration, were input into the LED array through a control program. The corresponding LEDs were driven at the specified levels, and the actual light power distribution was measured at the output plane, as shown in Fig. 4F to H. Figure 4F and G show a 3D surface plot and a 2D map of the light power distribution on the detection plane, respectively. Figure 4H shows the cross-sectional power distribution at the center of the *XY* plane (*X* = 0, *Y* = 0). The maximum irradiance measured in the experiment is 917.7μW/cm^2^, the minimum is 866.8μW/cm^2^, and the average value is 891.0μW/cm^2^. The discrepancy between the measured power values and simulation results is primarily attributed to the partial coherence of the light source, as incoherent superposition cannot perfectly simulate this process. The calculated INU of this configuration is 2.85%, with only a 0.6% deviation from the simulation result, thereby demonstrating the effectiveness of the homogenization method.

In another experimental situation requiring a longer illumination distance and a slightly smaller illumination area, the detector plane was positioned 100 mm from the light tube’s emission surface by adjusting the variable tube. Data were collected at 1-cm intervals within an 8cm×8cm area. Using the same approach to explore the optimal illumination under this configuration, we obtained optimized power distribution data for the detection plane. The simulation results are shown in Fig. 4I, with a nonuniformity value of 2.68%. The optimized configuration was then applied to the UV LED array, and the experimental results are shown in Fig. 4J, exhibiting a nonuniformity of 2.96%. To ensure the robustness of this method in practical lithography applications, for instance, its effectiveness at different proximity distances, we tested the nonuniformity within a relative deviation of 5 mm before and after the standard acquisition plane. Based on experimental testing and calculations, the nonuniformity values at 1-mm intervals from −5 to 5 mm were 3.78%, 3.58%, 3.82%, 3.54%, 3.36%, 2.96%, 3.25%, 3.19%, 2.81%, 3.19%, and 3.30%, respectively, as shown in Fig. 4K. The maximum and minimum values were 3.82% and 2.81%, respectively, with the value at the reference plane being 2.96%. The results indicate that despite a 5-mm deviation from the standard plane, far exceeding micrometer-level proximity distance (0 to 500 μm), the uniformity of the illumination surface does not degrade substantially, with the worst-case scenario still achieving over 96%. This confirms the excellent distance tolerance performance of the method. The system maintains high uniformity even under minor stage positioning errors, which is critical for industrial lithography stability.

In addition to characterizing the system’s illumination uniformity performance on the mask surface, we also measured the system’s energy utilization efficiency and divergence angle at the mask surface. Measurements of energy efficiency were performed at 2 representative exposure distances (5 and 100 mm from the exit surface of the variable square tube light guide to the detector plane) to evaluate distance-dependent efficiency variations. To ensure that the measured optical power remained within the resolution and dynamic range of the laboratory power meter, the No. 37 LED was illuminated at the second power level. The measured power and efficiencies are summarized in Table 1. Based on our testing of the light source, the light power incident on the mask surface increases linearly with the aperture setting. Due to the noncoherent nature of LEDs, we can represent the light power distribution across the mask surface illuminated by multiple/full-panel LEDs as the superposition of individual LEDs. Given this characteristic, the energy utilization efficiency for full-panel illumination can be approximated using tests conducted on a single LED. For specific measurement principles and procedures, refer to the corresponding sections in the Supplementary Materials.

**Table 1.** Energy efficiency of the system at 2 distances

Distance	*P*_exposure_ (𝜇W)	*P*_source_ (mW)	𝜂 (%)
100 mm	500.7	375.3	0.13
5 mm	1,289.8	375.3	0.34

The efficiency increases as the detector distance decreases from 100 to 5 mm, reflecting reduced propagation and scattering losses at shorter distances. Relative to widely adopted commercial systems, our present implementation is primarily intended as a proof of concept to validate feasibility, and its energy efficiency remains to be improved, but within the diffusion uniform illumination system, its energy utilization efficiency is considerable. Potential optimization pathways include the design of metasurfaces’ metallic layers and structures for specific wavelength ranges, surface coatings, enlarging the ADM area to capture and redistribute more light, and the incorporation of brightness enhancing films to increase energy utilization.

The measured divergence angles of the illumination at the mask plane after the square tube are $\theta_{x}=22.6^{\circ }$ and $\theta_{y}=18.7^{\circ }$, representing a substantial reduction compared with the native divergence angle of the LED source (65.3°). For details, refer to the Supplementary Materials section on the measurement principles and results of the system angular divergence. This demonstrates that the proposed ADM-based lensless monitoring and uniform illumination systems effectively confine the emission and improve angular uniformity. Nevertheless, there remains room for further optimization, particularly to reduce the slight asymmetry between the *x* and *y* directions and to approach an even tighter angular distribution.

To further validate the effectiveness of this system and method in actual lithography, we conducted experimental verification on our self-developed wafer-scale, large-field proximity lithography platform, as shown in Fig. 5B. This platform integrates a UV LED array light source with the light source *Z*-axis, ADMs, image-based overlay alignment measurement system, mask carrier, wafer displacement stage, monitoring software, and other components, with the illumination system being entirely free of lenses. As for the proximity lithography experiment, we employed a custom-patterned commercial mask (as shown in Fig. S5) of 3 mm thick, with a 7-inch square quartz glass plate covered with 100 nm of chromium (Cr). Custom patterns comprise line-pair patterns and grid patterns of varying dimensions centered in the 4 quadrants, along with triangular patterns distributed along the edges. The 6-inch silicon wafers were provided with the positive photoresist AZ5214 by spin-coating at 4,000 rpm for 30 s and subsequently baked for 100 s at 110°C. The wafers were exposed at 140, 150, 160, 170, and 180 mJ/cm^2^ doses under the proposed optimal configuration illumination system with the proximity distances from 0 to 50 μm. After exposure, the wafers were postexposure baked for 100 s at 120°C and developed in NMD developer for 150 s. Images of the developed patterns obtained using the microscopy (Olympus, Japan) are shown in Fig. 5C to H. The results of the 600-μm equilateral triangle at 140 mJ/cm^2^, the 70-μm grid at 140 mJ/cm^2^, the 30-μm line pair at 150 mJ/cm^2^, the 10-μm line pair at 140 mJ/cm^2^, the 10-μm line pair at 170 mJ/cm^2^, and the 2.5-μm line pair at 170 mJ/cm^2^ are illustrated in Fig. 5C to H, respectively. Different patterns of various critical dimensions can be printed with high clarity, demonstrating the effective application of the system and method proposed in this paper.

## Discussion

In summary, we propose a lithographic illumination system that integrates both monitoring and homogenization functions, enabled by ADMs. Notably, the system operates entirely without lenses and has been experimentally validated on a self-developed lithography platform to successfully expose patterns of varying sizes while simultaneously demonstrating its monitoring capability. In this work, the LED source and the diffuse reflection mechanism are employed not only to improve illumination uniformity but also to further decrease the spatial coherence of the illumination. The ADMs introduced in this work operate via a disorder engineering, incorporating both temporal and spatial statistical effects, and thus behaves as a stochastic modulation of the illumination. This mechanism differs fundamentally from conventional filter-based approaches, which rely on deterministic spatial filtering to shape the angular spectrum. By suppressing coherent interference in the near field, the proposed illumination scheme mitigates diffraction effects and improves tolerance to gap variations, without introducing a dominant penalty to the achievable resolution. The measured divergence angles remain sufficiently confined and do not constitute a dominant resolution-limiting factor. Different patterns of various critical dimensions (600-μm equilateral triangle, 70-μm grid, 30-μm line pair, 10-μm line pair, and 2.5-μm line pair) with the proximity distances from 0 to 50 μm have been printed with high clarity, demonstrating the effective application of the system and method proposed in this paper.

Compared with conventional systems based on MLAs or light integrator rods, our approach is simpler and more cost-effective, as it shifts complexity from expensive optical hardware to computational algorithms. By preacquiring calibration data and applying genetic algorithms, the LED array illumination is optimized to achieve high uniformity, while a GL-PLMA at the monitoring port evaluates LED states in real time. This enables fault identification and intelligent avoidance of malfunctioning LEDs in the illumination configuration, with nonuniformity suppressed to below 3%. The performance of the proposed lithographic illumination system prototype and its comparison with previous work are summarized in Table 2. Our system demonstrates distinct advantages in terms of a simple and compact optical architecture, lens-free design, and the integration of monitoring and homogenization. Conventional illumination systems typically rely on bulk refractive optics, diffusers, or micro-optical components fabricated by precision polishing, molding, or grayscale lithography. These approaches often involve complex fabrication processes, high material and assembly costs, maintenance requirements, and limited scalability, especially for large-area or highly compact illumination systems. In contrast, the proposed ADM-based system provides real-time monitoring and illumination homogenization through a compact, lens-free, planar architecture that is compatible with batch fabrication through industrial lithography. The approach is simpler and more cost-effective, as it shifts complexity from expensive optical hardware to computational algorithms. Furthermore, in conventional mercury-lamp- or LED-based illumination systems, a failure or degradation of individual light sources often necessitates system shutdown, light-source replacement, and subsequent mechanical or optical realignment, resulting in substantial maintenance costs and production downtime. In contrast, the proposed system incorporates a real-time monitoring algorithm that accurately tracks the operational status of each LED. Faulty LEDs can be promptly identified, and the optimization algorithm generates an equivalent uniform illumination configuration that excludes malfunctioning LEDs. This capability allows continuous operation without immediate hardware replacement or mechanical recalibration, thereby substantially reducing operational losses and maintenance-related downtime associated with partial light-source failures. This enables a more scalable and cost-effective solution. Besides, the fabrication of larger-area ADMs is expected to further enhance the system performance and fully exploit its architectural advantages. Relative to widely adopted commercial systems, our present implementation is primarily intended as a proof of concept to validate feasibility, and its energy efficiency remains to be improved, but within the diffusion uniform illumination system, its energy utilization efficiency is considerable. Potential optimization pathways include the design of metasurfaces’ metallic layers and structures, surface coatings, and the incorporation of brightness-enhancing films to increase energy utilization. Furthermore, our monitoring capability is not limited to the current demonstration, which focuses only on basic power-level detection. In future work, we plan to extend this functionality by leveraging large-scale data acquisition and artificial intelligence algorithms for rapid optimization and adaptive configuration under varying experimental conditions. In addition, the integration of light-shaping filter plates will be explored to enable advanced pupil-shaping control.

**Table 2.** Performance comparison of lithographic illumination systems

Reference	Source type	Nonuniformity	Exposure field	Optical components	Monitoring mechanism
[47]	I-line UHP	±2%	100mm×100mm	Flyeyes integrator + MLA + lens group ×5 + reflector ×2	N.A.
[9,12,48]	I-line UHP	±2.5%	D=150mm	MLA ×4 + lens group ×5 + filter plate	External
[49]	400 nm LEDs	±2.59%	200mm×200mm	MLA ×2 + lens group ×2	N.A.
[49]	Broadband UHP	±2.45%	200mm×200mm	MLA ×2 + lens group ×2	N.A.
[49]	I-line UHP	±2.9%	200mm×200mm	MLA ×2 + lens group ×2	N.A.
[50]	193 nm ArF laser	1.8%	D=150mm	MLA ×4 + lens group ×2 + filter plate	N.A.
[6]	355 nm laser	4.8%	D=220mm	Galvano scanner + eccentric rotation diffuser + lens group ×3	N.A.
[51]	405 nm fiber laser	4%	20.736mm×11.664mm	Homogenizing glass rod + sets of lenses + DMD + reflector	External
This work	I-line LEDs	<3%	100mm×100mm	ADMs	Integrated and unified

ADMs, asymmetric diffusion metasurfaces; DMD, digital micromirror device; MLA, microlens array; UHP, ultra-high-pressure mercury lamp

## Materials and Methods

The ADM samples were prepared on a 4-inch glass wafer (500 μm thickness) through a sequential multilayer aligned stepper photolithography process. Initially, a layer of photoresist was patterned by lithography to define a random patch array on the wafer. Subsequently, a 7-nm gold metallic film with the designed thickness was deposited across the entire wafer using electron-beam evaporation, covering both exposed regions and photoresist-protected areas. The resist was then stripped away in solvent, producing the first metallic patch layer on the wafer. Next, a 115-nm silica spacer was grown by plasma-enhanced chemical vapor deposition. A second photoresist layer was spin-coated and patterned by another lithography step, followed by deposition of a second metallic film. After lift-off of the photoresist, the second metallic patch layer remained. Finally, the whole structure was encapsulated with a 73-nm silica capping layer. The ADMs were subjected to 1,000 h of continuous UV irradiation (370 nm); no marked degradation in reflection/transmission characteristics was observed (ΔINU below 0.5%), confirming long-term stability for industrial use. Through quantitative theoretical calculations and analysis of the thermal expansion coefficients of the materials and structures used in the ADMs, as well as the phase shifts induced by temperature variations, it is determined that their numerical ranges are significantly smaller than manufacturing tolerances. The wavelength drift of LEDs with 15-nm half-widths and integrated heat dissipation is also limited. More importantly, ADMs can be designed for broadband response as required. By appropriately controlling the thickness and depth of the 2 unit metal layers, stable phase response across a very wide range can be ensured.

## Ethical Approval

Ethical approval was not required for this study, as it did not involve human participants, animal experiments, or any identifiable personal data.

## Data Availability

All essential data are provided within the manuscript and the Supplementary Materials, and further information is available from the corresponding authors on request.
