# Supplementary material for: Asymmetric Diffusion Metasurface-Based Lensless Monitoring and Uniform Illumination Systems for Optical Lithography
Source: Research (Wash D C). 2026 Mar 23;9:1197. doi: 10.34133/research.1197 (PMC13006733; doi:10.34133/research.1197)
Supplement: Supplementary 1 — Supplementary Text Figs. S1 to S5 Table S1 Movie S1 [file research.1197.f1.zip › Supplementary Materials.pdf]

Supplementary Materials for
Asymmetric diffusion metasurfaces based lensless monitoring and uniform illumination systems for optical lithography

Guangbiao Wang, Yanhua Shen, Yazhi Pi, Wenchao Kong, Yun Lai, Xu Ma, Yan Zhou*, Fan Li*, Zizheng Cao*, Lei Wang, Shaohua Yu*

*Corresponding author. Email: zhouy4295@outlook.com; lifan39@mail.sysu.edu.cn; zcaozju@zju.edu.cn; yush@cae.cn

This PDF file includes:

Supplementary Text

Figures S1 to S5

Table S1

Caption for Movie S1

Other Supplementary Materials for this manuscript:

Movie S1

Supplementary Text

Workflow and specifications for iteration parameters of the genetic algorithm

Fig. S1 presents the workflow of the genetic algorithm-based illumination configurations optimization procedure. The raw data for 64 LEDs at 16 power levels, collected on a $10\ \text{cm} \times 10\ \text{cm}$ plane, form the 1024 intensity matrices. Through selection, crossover, and mutation operations within the genetic algorithm, the global optimum is identified, yielding the corresponding LEDs configuration. As shown in Table S1, a population size of 1200 was adopted, with tournament selection (tournament size = 3), and elitism was applied by preserving 10% of the best individuals in each generation. Crossover was performed with a probability of 0.85 using a two-point scheme, while mutation was applied with a probability of 0.3 using uniform mutation, where each gene was altered within the range of 0 to 15 with a probability of 0.1. The maximum number of generations was set to 5000.

Characteristics of the UV LED array light source

Fig. S2 presents the characteristics of the UV LED array light source used in the experiment. The source consists of an 8×8 LEDs array with a pitch of 6 mm and an individual chip size of 6.5 mm, mounted on a 160 mm panel. Each LED can be controlled via serial communication, with 16 adjustable intensity levels. The system adopts air cooling for heat dissipation, as shown in Fig. S2A. The LEDs have a peak wavelength of 370 nm and a FWHM of 15 nm. Under the standard test conditions (ambient temperature: 25.3 °C, relative humidity: 65%), the operating current is 349.99 mA and the operating voltage is 6.81 V. The luminous intensity distribution curves of the LEDs are shown in Fig. S2B and Fig. S2C. In the C–G coordinate system, the measurement range is $T = C0\,°–180\,°$. The average luminous diffusion angles are $\theta(25\%) = 75.4\,°$, $\theta(50\%) = 65.3\,°$, and $\theta(75\%) = 48.8\,°$. The maximum luminous intensity is measured as $I_{\max} = 866.3$ mcd ($C = 0\,°$, $G = 7.5\,°$), while the on-axis intensity is $I_0 = 854.3$ mcd ($C = 0\,°$, $G = 0\,°$).

Principles and procedures for measuring system energy efficiency

We experimentally defined the system energy efficiency as Equation S1, where P_{source} is the emitted optical power from the LED source, and $P_{exposure}$ is the total optical power collected

within the effective area at the exposure plane.

$$\eta = \frac{P_{exposure}}{P_{source}} \times 100\%, \tag{S1}$$

P_{source} was calculated from the measured radiant power density at the LED proximal position. A calibrated UV power detector with a 10-mm aperture fully covering the emitted spot was used to measure the radiant power density I_{source} (in mW/cm^2). The total emitted power was calculated as Equation S2, where $A_{detector} = \pi(D/2)^2$ is the detector aperture area and D = 10 mm.

$$P_{source} = I_{source} \cdot A_{detector}, \tag{S2}$$

To obtain $P_{exposure}$, the exposure plane (100 mm × 100 mm) was discretized into a regular 10-mm grid. The optical power P_i was measured at the center of each grid cell using the same calibrated power meter, and the total power was obtained by numerical integration as Equation S3:

$$P_{exposure} = \sum_{i}^{N} P_i \cdot \Delta A, \tag{S3}$$

where P_i denotes the measured optical power at the i-th sampling position and $N = 100$, $\Delta A = 1$ cm^2 is the area of each grid cell.

Principles and results for measuring system angular divergence

The angular divergence of the illumination after the variable square-tube was characterized based on spatially resolved power measurements at the mask plane. Due to the extended square exit aperture (140 mm × 140 mm) and the multiple diffuse reflections inside the square tube, the emitted light cannot be treated as a point source. Therefore, an effective divergence angle was defined based on the spatial intensity distribution rather than a central ray approximation. The schematic diagram of the test system is shown in Fig. S3. The optical power distribution was measured at the mask plane located 100 mm away from the square tube exit. Power measurements were performed by scanning a calibrated UV power detector along the horizontal (x) and vertical (y) directions with a spatial step size of 1 mm. The measured power values were normalized to the maximum recorded intensity in each position. For each direction, the effective half-divergence angle was defined as the angular offset corresponding to the positions where the normalized intensity decreased to 0.5 of the maximum value on both sides of the beam footprint. The angular coordinates were obtained

by geometrically converting the measured spatial offsets Δx or Δy at the mask plane into angles according to Equation S4

$$\theta = arctan(\frac{\Delta s}{L}), \tag{S4}$$

where $\Delta s = \Delta x$ or Δy denotes the lateral displacement from the optical axis and L = 100 mm is the propagation distance from the square tube exit to the mask plane. The divergence angles on each side were calculated independently, and the overall divergence was reported as the sum of the left and right half-angles for each axis.

The measured intensity profiles along the x and y directions are shown in Fig. S4. The corresponding full width at half maximum (FWHM) values are 180 mm in the x direction and 173 mm in the y direction, respectively. Along the x direction, the half-maximum intensity positions were located at lateral offsets of 20 mm and 20 mm, respectively. Substituting these values into Equation S4 yields Equation S5:

$$\theta_x = arctan(\frac{20}{100}) + arctan(\frac{20}{100}) = 11.3° + 11.3° = 22.6°, \tag{S5}$$

Similarly, along the y direction, the corresponding offsets were measured to be 16 mm and 17 mm, resulting in Equation S6:

$$\theta_y = arctan(\frac{16}{100}) + arctan(\frac{17}{100}) = 9.1° + 9.6° = 18.7°, \tag{S6}$$

Custom patterns of the mask

Fig. S5 presents a 7-inch mask designed with a 4-inch circular active region at the center, which is divided into four quadrants. Each quadrant contained four types of test patterns: line pairs, grid structures, overlay alignment marks, and other functional features. A triangular pattern is placed along the upper edge of the region. Specifically, the red-boxed area corresponds to an equilateral triangle with a side length of 600 μm. The blue-boxed region includes line-pair structures with linewidths ranging from 1 μm to 30 μm, whereas the green-boxed region contains grid patterns with feature sizes between 2 μm and 80 μm.

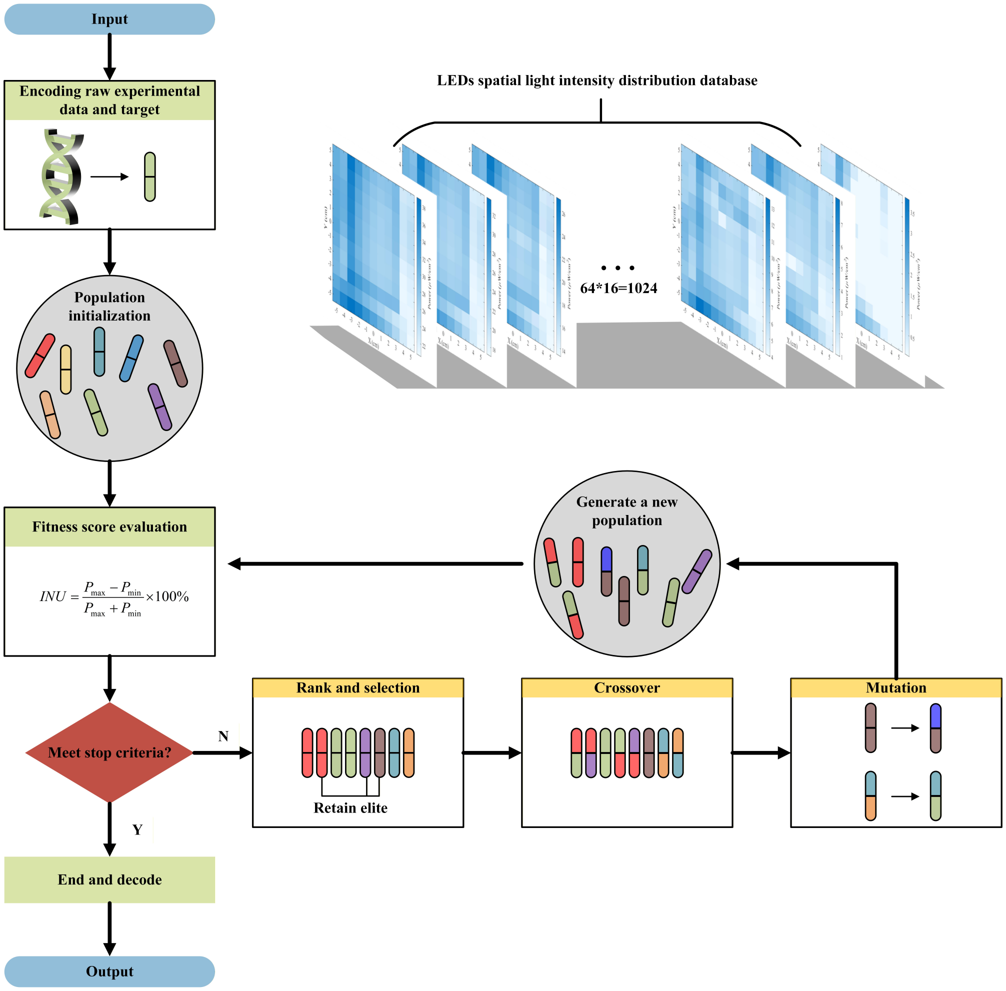

Figure S1: **Workflow of the genetic algorithm-based illumination configurations optimization procedure.** The raw data for 64 LEDs at 16 power levels, collected on a 10 cm × 10 cm plane, form the 1024 intensity matrices. Through selection, crossover, and mutation operations within the genetic algorithm, the global optimum is identified, yielding the corresponding LEDs configuration.

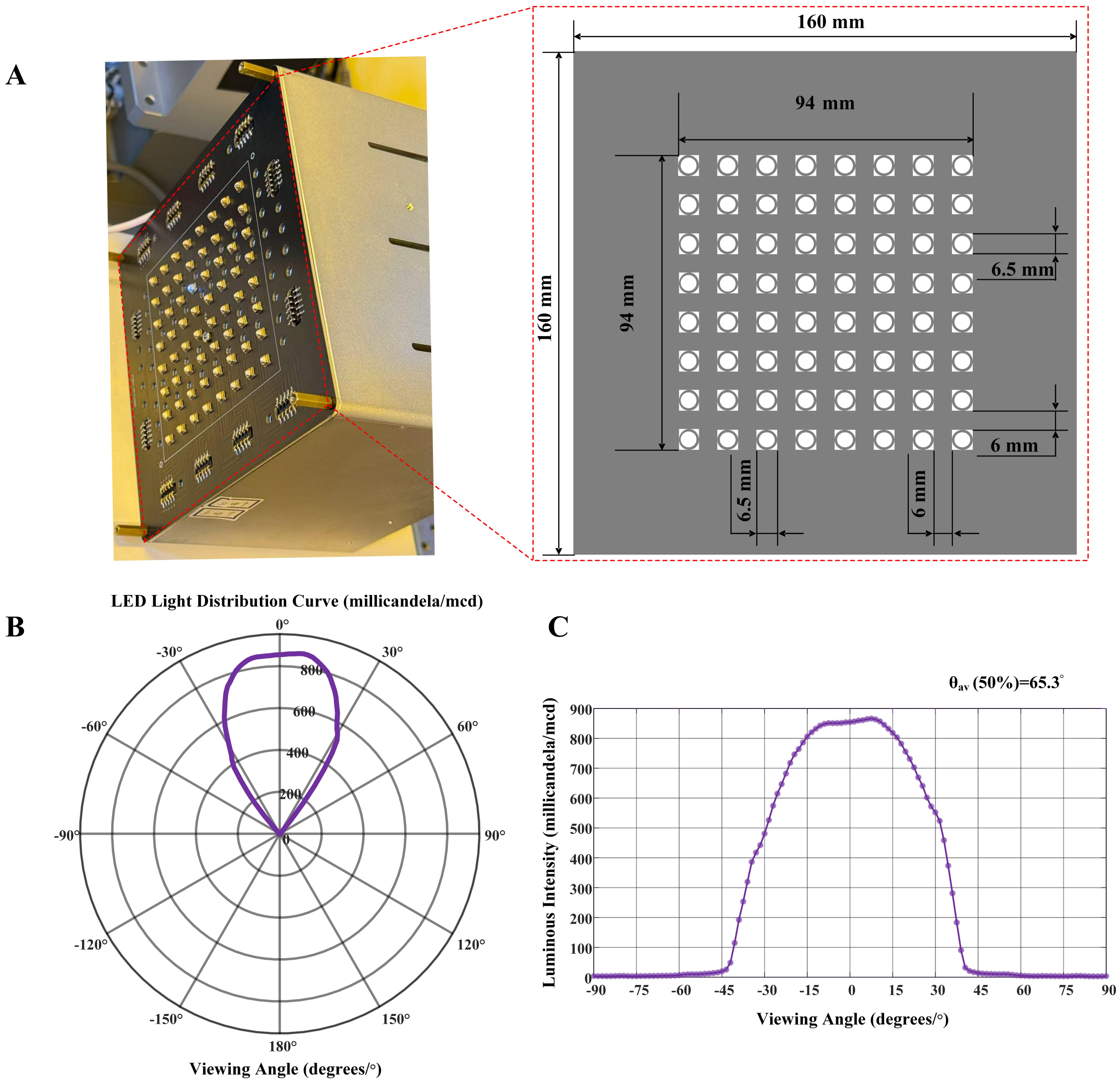

Figure S2: **Photograph and characteristics of the UV LED array light source used in the experiment.** (**A**) LEDs layout and dimensional parameters of the light source. The source consists of an 8 × 8 LEDs array with a pitch of 6 mm and an individual chip size of 6.5 mm. (**B**) Luminous intensity distribution curve of the LED. The maximum luminous intensity is measured as I_{max} = 866.3 mcd (C = 0 °, G = 7.5 °), while the on-axis intensity is I_0 = 854.3 mcd (C = 0 °, G = 0 °). (**C**) The average luminous diffusion angles are θ(25%) = 75.4 °, θ(50%) = 65.3 °, and θ(75%) = 48.8 °.

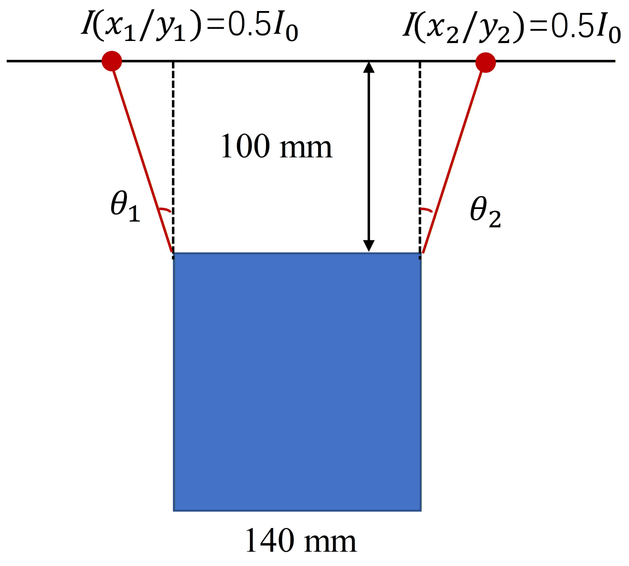

Figure S3: **Schematic of the angular divergence experimental measurement system.** The square tube features a 140 mm × 140 mm output aperture, and measurements are performed at the mask plane located 100 mm from the exit with a spatial step size of 1 mm.

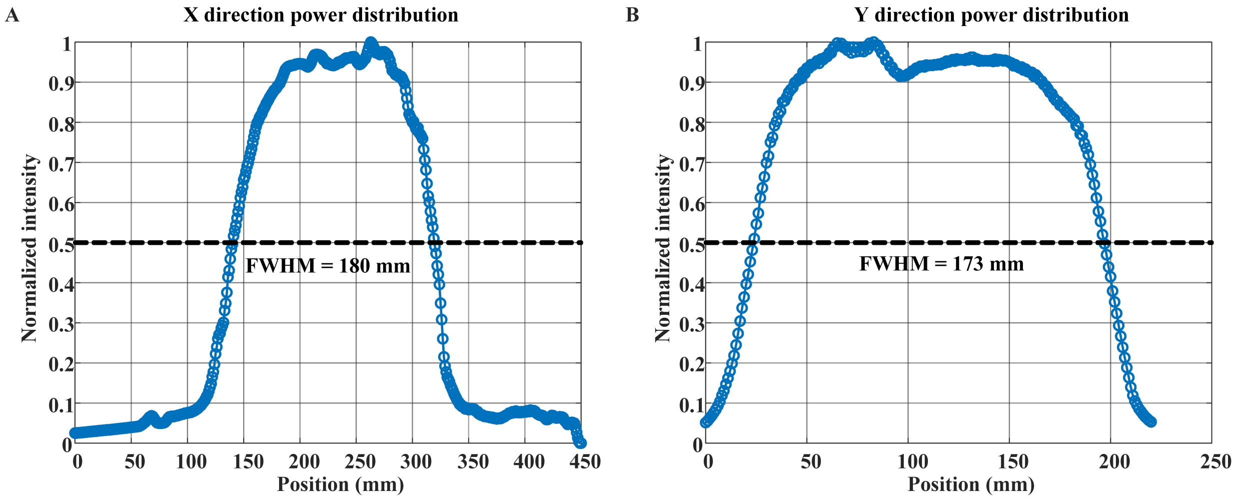

Figure S4: **Measured intensity profiles at the mask plane used for divergence angle extraction in the x and y directions.** (**A**) Intensity distribution along the *x* direction with a FWHM of 180 mm. (**B**) Intensity distribution along the *y* direction with a FWHM of 173 mm.

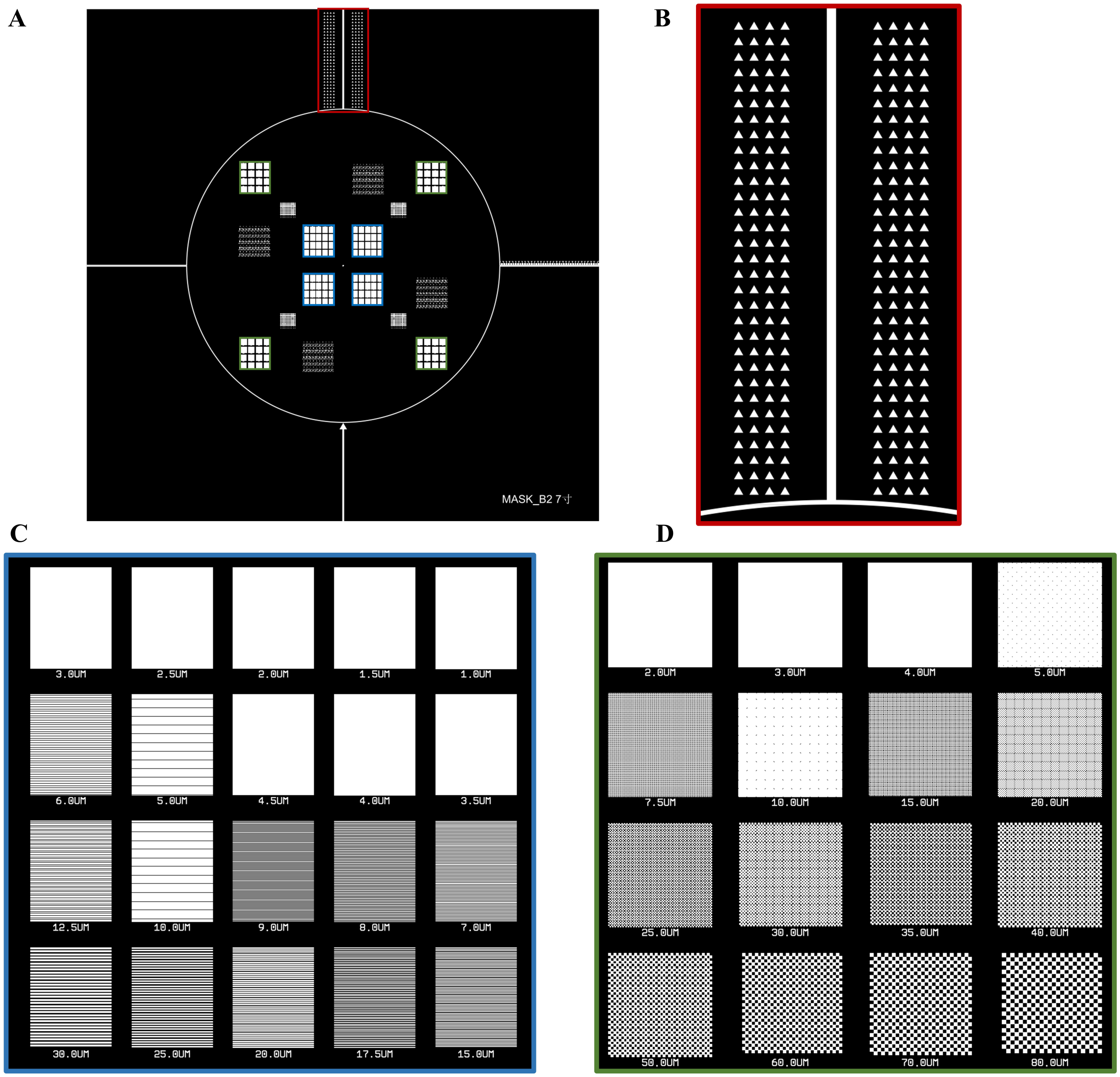

Figure S5: **Layout of the 7-inch mask.** (**A**) The central 4-inch circular area is divided into four quadrants, each containing four types of test patterns: line pairs, grid structures, alignment marks, and functional features. A triangular pattern is placed at the upper edge. (**B**)The red box highlights an equilateral triangle with a side length of 600 μm. (**C**)The blue and green boxes (**D**) indicate line-pair (1 μm to 30 μm) and grid (2 μm to 80 μm) patterns, respectively.

Table S1: **Specifications for iteration parameters of the genetic algorithm.**

Parameter	Specification
Variable type	Matrix indices
Population size	1200
Selection options	Tools.selTournament, 3
Elite size	120
Crossover operator	Tools.cxTwoPoint
Crossover fraction	0.85
Mutation fraction	0.3
Mutation options	Tools.mutUniformInt, [0,15], 0.1
Max generations	5000

Caption for Movie S1. Operation of the UV-LED intensity acquisition system. This video demonstrates the operation of the UV-LED illumination intensity acquisition system. The system follows an S-shaped scanning path to sequentially collect data from 64 LEDs at 16 intensity levels. By sequentially controlling each LED, intensity data at every sampling position are recorded. The collected data are then processed to reconstruct the complete illumination profile for each LED on the sampling plane. This scanning strategy minimizes measurement errors induced by long-range motion and repeated repositioning.
